# Sneezing during Micturition: A Possible Trigger of Acute Bacterial Prostatitis

**DOI:** 10.1155/2015/626409

**Published:** 2015-08-18

**Authors:** William Derval Aiken

**Affiliations:** Department of Surgery, Radiology, Anaesthesia and Intensive Care, Section of Surgery, Division of Urology, Faculty of Medical Sciences, University of the West Indies, Mona, Kingston 7, Jamaica

## Abstract

A perfectly well 39-year-old man sneezed during micturition and developed classic features of acute bacterial prostatitis corroborated by laboratory evidence of prostatic inflammation/infection. The prostate-specific antigen level at presentation was 9.6 ng/mL and declined to 1.23 ng/mL one month later on levofloxacin. This is the first report in the medical literature of sneezing while voiding being a possible trigger of acute bacterial prostatitis. A biologically plausible mechanism is provided.

## 1. Introduction

Several mechanisms of development of acute bacterial prostatitis (ABP) are either known or postulated to occur. ABP is a recognised complication of transrectal prostate biopsy and occurs due to prostatic inoculation with large bowel organisms. Ascending infection facilitated by incomplete bladder emptying and urethral instrumentation may involve the prostate through intraprostatic urinary reflux, which empirically has been demonstrated to occur [1]. Other suggested routes of prostatic involvement include haematogenous and lymphatogenous spread as well as direct spread from pelvic foci of infection.

Described below is a man who was perfectly well until he sneezed during micturition and developed classic features of ABP. This constellation of events triggering ABP has not been previously reported in the medical literature. A brief description of the postulated mechanisms is provided.

## 2. Case Presentation

A perfectly well 39-year-old man with no known chronic illnesses or prior history of lower urinary tract disorder/disease arrived home and went to urinate. While urinating he sneezed which was accompanied by lancinating perineal pain that was short-lived, and he was able to complete micturition normally. There was no haematuria or urethral bleeding noted. Later that day he developed urgency, urinary frequency (every 2-3 hours), and nocturia (3 episodes/night) and burning and pain on micturition accompanied by fever, chills, rigors, headache, and malaise. Three days later he developed terminal haematuria and sought medical attention.

When seen, he denied having any flu-like symptoms or urethral discharge. He appeared unwell and had a fever of 99.6 Fahrenheit. His mucous membranes were pink and moist and the general physical examination was normal. His pulse rate was 103/minute and BP 130/73 mmHg. The abdominal examination was normal and he had no renal angle or suprapubic tenderness. Examination of the genitalia was normal except for tenderness in the perineum. Digital rectal examination (DRE) revealed a tender boggy prostate. The International Prostate Symptom Score (IPSS) and QoL score were 13 and 5 (“Unhappy”), respectively.

Dipstick urinalysis revealed 2+ blood and protein and positive nitrite. Urine microscopy demonstrated 4+ bacteria, 20–60 white blood cells, and 5–10 red blood cells/high power field (hpf). After obtaining a mid-stream specimen of urine for culture and sensitivity, he was started on empirical levofloxacin 500 mg once daily, nimesulide, a nonsteroidal anti-inflammatory drug (NSAID), one tablet twice daily, and controlled release tamsulosin 0.4 mg nightly. Urine culture demonstrated >10^5^ colony forming units/mL of* Morganella morganii*. The white blood cell count was 14 × 10^9^/L, with an absolute and relative neutrophilia and an erythrocyte sedimentation rate (ESR) of 35/hour. The serum prostate-specific antigen (PSA) was 9.6 ng/mL.

The IPSS and QoL score declined to 9 and 4 (“Mostly dissatisfied”), respectively, 48 hours after initiating treatment; a further decline to 6 and 3 (“Mixed”), respectively, was seen 1 week later. When reviewed 2 weeks later the IPSS was 5 and the QoL score remained at 3. His fever disappeared 24 hours after the initial visit and the urgency, urinary frequency, and pain and burning on micturition abated 3 days after the doctor's visit. He complained of ejaculatory discomfort and heightened perineal sensation when sitting for long periods. On examination he had perineal tenderness on deep palpation. His temperature was 98.3°F, pulse rate 88/minute, and BP 130/90 mmHg. Repeat urinalysis was completely normal and repeat urine culture returned “No growth.” The WBC was now normal at 8.9 × 10^9^/L and the PSA declined to 3.04 ng/mL.

**Figure 1:**
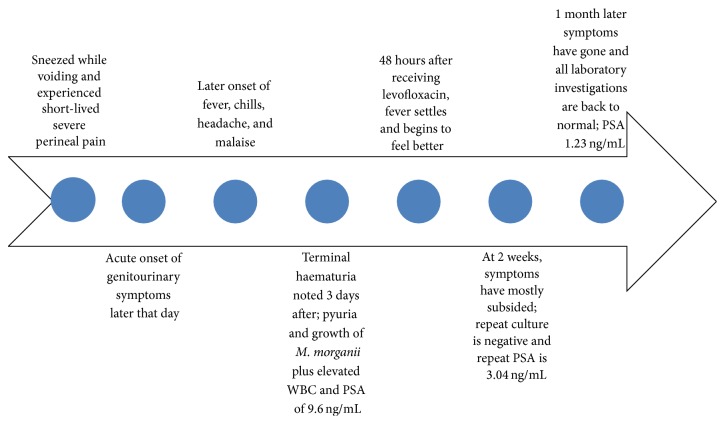
Time course of events after sneezing while voiding.

One month after presentation the patient's ejaculatory and perineal discomfort had resolved and he denied having any reduction in the strength of urine flow. The pulse rate was 77/minute, BP 130/82 mmHg, and temperature 98.3°F. Repeat DRE demonstrated a small nontender prostate of normal consistency. Repeat urinalysis was normal and PSA returned at 1.23 ng/mL (Figure 1).

After four-month follow-up the patient has remained free of any new-onset storage or voiding lower urinary tract symptoms (LUTS), denies having any ejaculatory or postejaculatory pain or discomfort, has no perineal or pelvic pain or discomfort, and appears to be completely devoid of any long term sequelae. Repeat PSA 4-months after initial presentation is now 0.69 ng/mL.

## 3. Discussion

When sneezing occurs during bladder storage there is an abrupt increase in the urethral closure pressure due to increased efferent somatic motor activity in the pudendal nerves causing contraction of the external urethral sphincter (EUS) and levator ani muscles, thereby preventing urinary incontinence [2, 3]. However, during voiding there is normally relaxation of the EUS, the pelvic floor, and bladder neck immediately prior to and throughout voiding until it is completed [4].

When the patient sneezed during voiding his bladder outlet and pelvic floor would have initially been in an open and relaxed state. It is theorized that the sneeze reflex would have overridden the voiding reflexes, due to the integration of these reflexes by the periaqueductal grey in the brainstem [5]. This would have resulted in reflex contraction of the EUS and pelvic floor causing an abrupt obstruction to urine flow during voiding. Simultaneous with this abrupt urethral obstruction would have been a precipitous increase in intravesical pressure (Pves) arising from the transmission of the sudden increase in intra-abdominal pressure (IAP), due to sneezing, to the already contracting urinary bladder. It is not known whether the bladder neck would have simultaneously closed with the EUS, but whether it remained open or not during sneezing, an abrupt and severe pressure-head in the urine-filled prostatic urethra upstream of the contracted EUS would have been generated.

The abruptly increased pressure head in the urine-filled prostatic urethra would have driven urine into the prostatic ducts that open directly into the prostatic urethra [6, 7]. The more horizontally oriented ducts draining the posterior zone of the prostate [8] would have encountered potentially greater intraprostatic urinary influx and possible denudation of the epithelial cells lining the ducts and acini from the shearing force of the urine gushing into them under high pressure.

An initial chemical prostatitis from the constituents of the sterile urine would have likely ensued. With the denudation of the prostatic ducts and acini, epithelial barriers to infection would have been compromised, creating an environment conducive to secondary infection [9]. The evolution of the patient's symptoms after the inciting event (Figure 1) and infection with* Morganella morganii*, a facultative opportunistic gram negative bacillus that is part of the faecal flora, is consistent with this hypothesis.

A compelling argument for a possible cause-effect relationship between the sneeze while voiding and ABP has been presented and is based on the time course of events and corroboration by laboratory evidence of prostatitis and UTI. A biologically plausible mechanism for the relationship has been presented.
